# A review on plant-derived natural products and their analogs with anti-tumor activity

**DOI:** 10.4103/0253-7613.41038

**Published:** 2008

**Authors:** K.K. Dholwani, A.K. Saluja, A.R. Gupta, D.R. Shah

**Affiliations:** A.R College of Pharmacy affiliated to Sardar Patel University, Vallabh Vidyanagar, Dist. Anand, Gujarat - 388 120, India; 1 Maliba Pharmacy College affiliated to Veer Narmad South Gujarat University, Gopal Vidyanagar, Tarsadi, Ta-Bardoli, Dist-Surat, Gujarat - 394 350, India

**Keywords:** Anticancer activity, antitumor compounds, plant-derived natural products

## Abstract

Traditional medicines, including Chinese herbal formulations, can serve as the source of potential new drugs, and initial research focuses on the isolation of bioactive lead compound(s). The development of novel plant-derived natural products and their analogs for anticancer activity details efforts to synthesize new derivatives based on bioactivity- and mechanism of action-directed isolation and characterization coupled with rational drug design - based modification. Also, the anticancer activity of certain natural products and their analogs can be enhanced by synthesizing new derivatives based on active pharmacophore models; drug resistance and solubility and metabolic limitations can be overcome by appropriate molecular modifications; and new biological properties or mechanisms of action can be added by combining other functional groups or molecules. Preclinical screening for *in vitro* human cell line panels and selected *in vivo* xenograft testing then identifies the most promising drug development targets.

There are various classes of recently discovered compounds that possess potent antitumor activity. These compounds were obtained by bioactivity- and mechanism of action-directed isolation and characterization, coupled with rational drug design-based modification and analog synthesis. Research highlights include GL331, which is currently in anticancer clinical trials.

Historically, numerous useful drugs have been developed from lead compounds originally isolated from medicinal plants.[1] Three main research approaches are used in this drug discovery and development process: (1) bioactivity- and mechanism of action-directed isolation and characterization of active compounds, (2) rational drug design - based modification and analog synthesis, and (3) mechanism of action studies. Traditional medicines, including Chinese herbal formulations, can serve as the source of potential new drugs and initial research focuses on the isolation of bioactive lead compound(s). Next, chemical modification is attempted with the aim of increasing activity, decreasing toxicity, or improving other pharmacological profiles. Preclinical screening in the National Cancer Institute (NCI) *in vitro* human cell line panels and selected *in vivo* xenograft testing then identifies the most promising drug development targets. Four types of studies help refine the active structure:

1. Structure-activity relationship (SAR) studies, including qualitative and quantitative SAR.
2. Mechanism of action studies, including drug-receptor interactions and specific enzyme inhibitions.
3. Drug metabolism studies, including identification of bioactive metabolites and blocking of metabolic inactivation.
4. Molecular modeling studies, including determination of three-dimensional pharmacophores.

The preclinical development of bioactive natural products and their analogs as chemotherapeutic agents is a major objective of this kind of research program. Drug development then addresses toxicological, production, and formulation concerns before clinical trials can begin.

The following sections describe the research in the development of various anticancer lead compounds. In this section, the development of etoposide-related anticancer compounds details the efforts to enhance activity by synthesizing new derivatives based on active pharmacophore models; to overcome drug resistance, solubility, and metabolic limitations by appropriate molecular modifications; and to combine other functional groups or molecules to add new biological properties or mechanisms of action. The clinical trials of GL331, an etoposide analog, attest to the feasibility and success of this strategy.

## Antitumor Agents - Novel Plant Cytotoxic Antitumor Principles and Analogs

Since 1961, nine plant-derived compounds have been approved for use as anticancer drugs in the US: vinblastine (Velban), vincristine (Oncovin), etoposide (VP-16, **1**), teniposide (VM-26, **2**), Taxol (paclitaxel), navelbine (Vinorelbine), taxotere (Docetaxel), topotecan (Hycamtin), and irinotecan (Camptosar). The last three drugs were approved by the Food and Drug Administration in 1996.

## Novel Antitumor Etoposide Analogs

The synthesis and biological evaluation of etoposide derivatives has been a primary research for many years. Some highlights of this research follow and serve to illustrate several aspects of the drug development process.

Etoposide (**1**) and its thiophene analog teniposide (**2**) are used clinically to treat small cell lung cancer, testicular cancer, leukemias, lymphomas, and other cancers[2–5]; however, problems such as myelosuppression, drug resistance, and poor bioavailability limit their use and necessitate further structural modification.[6] Etoposide is structurally related to the natural product podophyllotoxin (**3**), a bioactive component of *Podophyllum peltatum*, *P. emodi,* and *P. pleianthum*, but not etoposide, binds reversibly to tubulin and inhibits microtubule assembly.[7] Etoposide inhibits the enzyme DNA topoisomerase II (topo II) and, subsequently, increases DNA cleavage.[7] Furthermore, with **1**, biooxidation to an E-ring *ortho-*quinone results in covalent binding to proteins[8,9] and hydroxyl radicals formed by metal-etoposide complexes cause metal- and photo-induced cleavage of DNA.[10]

## 4-Amino-etopodophyllotoxin Derivatives Including Gl331

Several series of 4-alkylamino and 4-arylamino epipodophyllotoxin analogs are synthesized starting from the natural product podophyllotoxin (**3**).[11] Computer modeling studies show that the amino group does not significantly alter the molecular conformation and that bulky groups are tolerated in the C-4 position. Compared with etoposide (**1**), several compounds showed similar or increased percentage inhibition of DNA topo II activity and percentage cellular protein-DNA complex formation (DNA breakage) [Table 1]. However, the most exciting finding is the increased cytotoxicity of these derivatives in **1**-resistant cell lines [Table 2]. GL331 (**4**),[12] which contains a *p*-nitroanilino moiety at the 4β position of **1**, has emerged as an excellent drug candidate. It has been patented by Genelabs Technologies, Inc. and has completed phase I clinical trials as an anticancer drug at the M.D. Anderson Cancer Center. Like **1**, GL331 functions as a topo II inhibitor, causing DNA double-strand breakage and G2-phase arrest. GL331 and **1** causes apoptic cell death, inhibiting protein tyrosine kinase activity (both compounds) and by stimulating protein tyrosine phosphatase activity and apoptotic DNA formation (GL331).[13] Compared with **1**, GL331 has several advantages: (1) it shows greater activity both *in vitro* and *in vivo*; (2) its synthesis requires fewer steps, leading to easier manufacture; and (3) it can overcome multidrug resistance in many cancer cell lines (eg, KB/VP-16, KB/VCR, P388/ADR, MCF7/ADR, L1210/ADR, HL60/ADR, and HL60/VCR).[12] Formulated GL331 shows desirable stability and biocompatibility and a pharmacokinetic profile similar to that of **1**.[14] Initial results from phase I clinical trials[14] in four tumor types (nonsmall cell and small cell lung carcinoma, colon cancer, and head/neck cancers) showed marked antitumor efficacy. Side effects were minimal, with cytopenias being the major toxicity. Maximum tolerated dose (MTD) was declared at 300 mg/m^2^. In summary, GL331 is an exciting chemotherapeutic candidate with a novel mechanism of action, predictable and tolerable toxicity, and evidence of activity in refractory tumors. A phase IIa clinical trial against gastric carcinoma has been initiated. This compound is one illustration of successful preclinical drug development from the research program.

**Table 1:** Mechanistic screening assays

	*Compound AM*	*ID_50_ (μM) tubulin polymerization*	*% Inhibition of tubulin at 100 μM*	*% Protein-linked DNA breaks*	*IC_30_ (μM) for maximal DNA breaks*
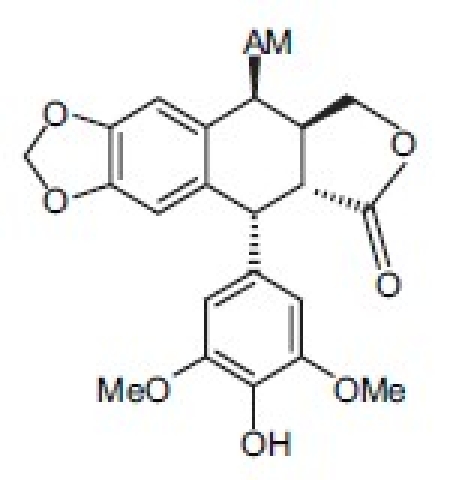
	Etoposide (1)	>100	0	100	10
	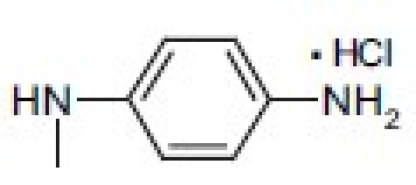	10	88	100	2
	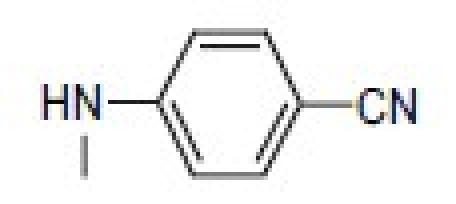	>100	34	125	6
	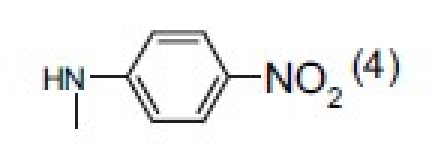	>100	35	140	2
	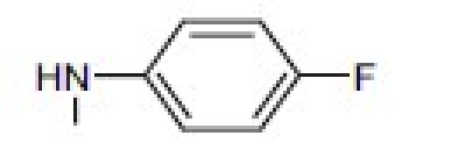	50	60	141	5
	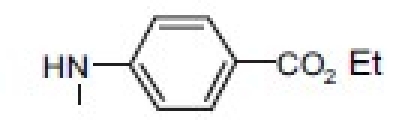	100	50	131	5
	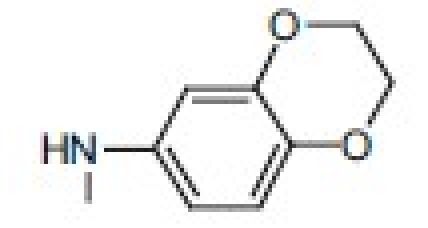	5	86	110	6
	Podophyllotoxin(3)	0.5	100	ND	ND

ND: Not determined

**Table 2:** Cytotoxicity assays against KB cells and resistant variants

	*Compund AM*	*ID_50_ (μM)*			
		
		*KB ATCC*	*KB IC*	*KB 7D*	*KB 50*
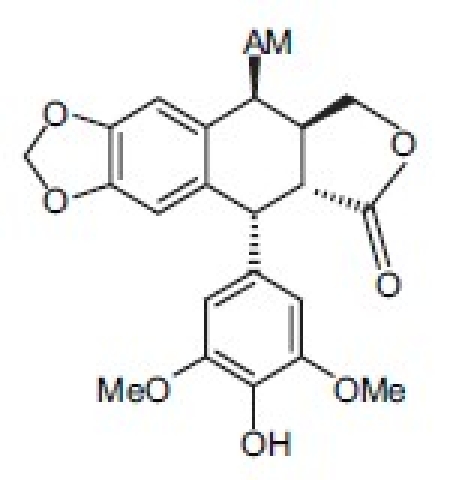
	Etoposide (1)	0.60	0	100	10
	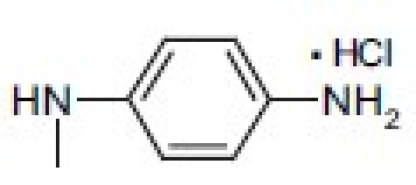	0.59	88	100	2
	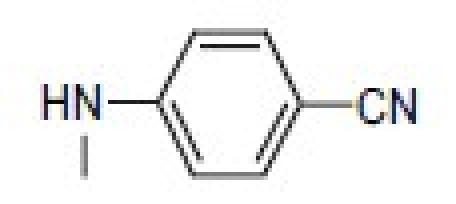	0.61	34	125	6
	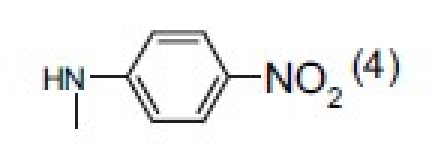	0.49	35	140	2
	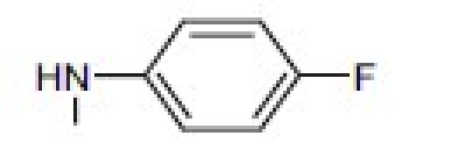	0.67	60	141	5
	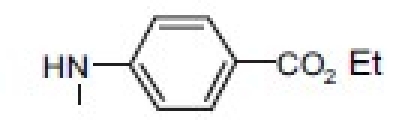	0.84	50	131	5
	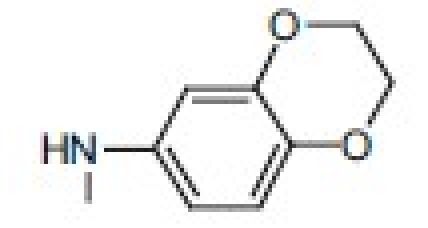	0.68	86	110	6

## γ-Lactone Ring-modified 4-Amino etoposide Analogs

**Figure 1:**
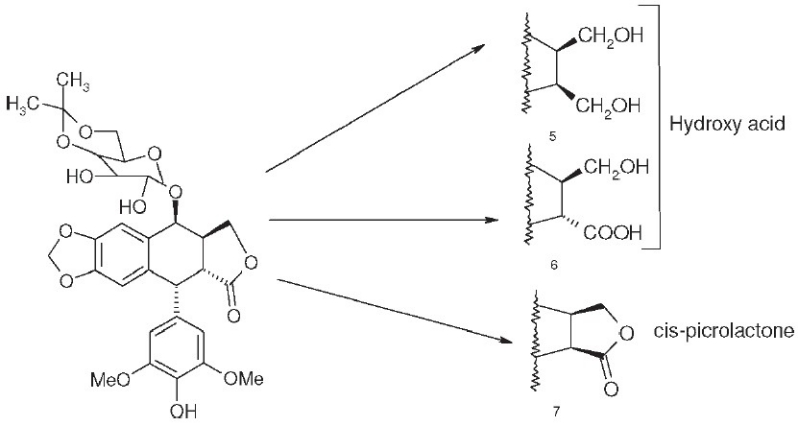
Metabolism of etoposide to inactive species

Metabolism of etoposide (**1**) [Figure 1] causes its inactivation by hydrolysis to the inactive *cis*- (**5**) and *trans*- (**6**) hydroxyl acids and epimerization to the cis-picro-lactone (**7**). To overcome this deficiency, lactone carbonyl was replaced with a methylene group, generating new γ-lactone ring-modified 4-amino epipodophyllotoxins.[15] The unsubstituted- (**8**) and *p*-fluoro- (**9**) anilino compounds showed topo II inhibition (ID_50_ = 50 µM) and DNA breakage (125 and 139%, respectively, at 20 µM) equal to and greater than those of **1** (50 µM and 100%, respectively).[15]

## Podophenazine Derivatives As Novel Topo Ii Inhibitors

**Figure 2:**
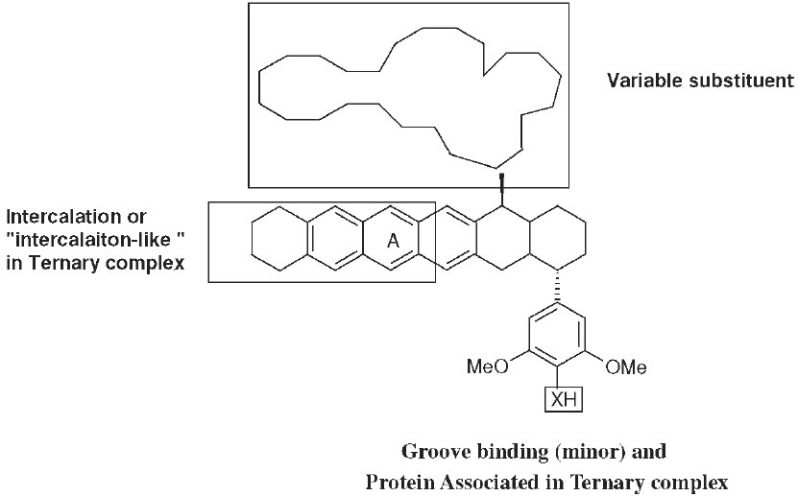
MacDonald's composite pharmacophore model of 1-like analogs

Another area of modification is the methylenedioxy ring of etoposide (**1**). MacDonald *et al*.[16] have proposed a composite pharmacophore model for **1**-like analogs that express topo II activity [Figure 2]. In this model, an intercalation or ‘intercalation-like’ domain includes the methylenedioxy ring. Furthermore, CoMFA steric contour plots of DNA-**1** complexes show an active and sterically favorable area of interaction in this same region.[17] Accordingly, we synthesized and evaluated podophenazine derivatives (**10** and **12**) of our 4β-amino substituted **1**-analogs. In these analogs, a quinoxaline heteroaromatic ring system replaces the methylenedioxy ring; thus, the planar aromatic area extends further into the ‘intercalation’ domain of MacDonald's model. Compared with **1**, the unsubstituted (**10**) and a di-chlorinated (**11**) podophenazine showed comparable and greater cytotoxicity against **KB** and **1**-resistant KB-7D cells, respectively [Table 3]. However, these compounds do not stimulate DNA breakage and, thus, their mechanism of topo II inhibition is distinct from that of **1** and its congeners.[17,18]

**Table 3:** Cytotoxicity and topo II inhibitory activity of podophenazines 10 to 12

*Compound*	*IC_50_ (μM)*		*Fold-stimulation of protein-linked DNA breaks*	
		
	KB	KB-7D	50 μM	100 μM
1	0.16	24	24.6	28.4
10	0.11	0.56	3.1	4.5
11	0.48	10.59	1	1
12	6.63	ND	ND	ND

ND: Not determined

## Etoposide Analogs with Minor Groove-binding Enhancement

The CoMFA study mentioned above also revealed that the steric and electronic fields of the 4-*0*′-demethylepipodophyllotoxins are compatible with the stereochemical properties of the DNA backbone. Thus, an increase in the minor groove binding ability of our 4-amino-epipodophyllotoxin analogs should increase topo II inhibition. We linked two known minor groove-binding functional groups, which are structural components of the cytotoxic polypeptide netropsin, to a *p-*aminoanilino epipodophyllotoxin through an amide bond.[19] The new compound (**13**), with a 1-methyl-4-nitro-2-pyrrolecarboxyl group, showed potent cytotoxicity with log GI_50_ values less than −8 in MOLT-r leukemia and MCF-7 breast cancer cell lines; the corresponding values of etoposide (**1**) were −5.99 and −5.36, respectively. Increased cytotoxicity was also found in KB cells (ID_50_ /LD_50_:**13**, 0.04/0.15;**1**, 0.2/3.0 µM) with a lower-fold increase in etoposide-resistant KB-7D cells (ID_50_/LD_50_:**13, 0.2/0.25;1**, 25/not determined, µM). Inhibitory activity against topo II was also greater with a lower IC_100_ for topoisomerase II inhibitory activity (**13**, 12.5;**1**, 100 µM) and greater percentage inhibition of protein-linked DNA breaks (**13**, 225%; **1**, 100%) at 12.5 µM.

## Dual Topo I and Topo Ii Inhibitors

Topoisomerase II inhibitors (such as etoposide, **1**) and topoisomerase I inhibitors (such as the antitumor natural product camptothecin, **16**) are useful in cancer chemotherapy. Their cytotoxicity results from the inhibitor's interaction with and stabilization of the enzyme-DNA cleavable complex. Other compounds, such as the 7-*H-*benzopyrido[4,3-*b*]indole derivative inotoplicine, simultaneously inhibit both enzymes and, thus, may circumvent topoisomerase-mediated drug-resistance mechanisms. Therefore, two potential dual inhibitors, **14** and **15**, were synthesized by chemically linking a *p*-aminoanilino- and an *o*aminoanilino-substituted epipodophyllotoxin, respectively, with 4-formyl camptothecin through an imine bond.[20] The growth-inhibitory properties of these new compounds closely resembled the behaviors of both the topo I- and topo II-inhibitory components. Compared with **1** and GL331 (**4**), **14** and **15** were more cytotoxic in several cancer cell lines, including HOP-62 leukemia, SW-620 colon cancer, MCF/ADR adriamycin-resistant breast cancer, and A-498 renal cancer [Table 4]. In addition, when cytotoxicity was measured in KB and drug-resistant KB-variants, **14** and **15** showed a lower-fold decrease in cytotoxicity (approximately 2-fold and 6-fold) than did **1** (80-fold) and **16** (30-fold) in **1**-resistant (KB-7D) and **16**-resistant (KB-CPT) cell lines, respectively. Both conjugate compounds also showed a lower-fold decrease in a vincristine-resistant cell line (KBVCR) than did **1** [Table 5]. Compound **15**, especially, showed low *in vivo* toxicity when given i.p. to nude mice. The compounds also stimulated DNA cleavable complex formation with both topo I and topo II. Both compounds had about 2-fold lower activities than **16** in the former assay. In the latter assay, **15**, but not **14**, was as active as, in general, conjugation resulted in cleavable complex-forming dual topoisomerase inhibitors with cytotoxic activity against drug-resistant cells. This type of compound is worthy of further development into clinically useful anticancer drugs.

**Table 4:** Selected data from the NCI human tumor cell line panel for 14 and 15

*Cell line*	*Log GI_50_ (M)*			
	
	*14-HCl*	*15-HCl*	*1*	*4*
HOP-62	<−8.00	−8.07	−3.85	−6.5
SW-620	<−8.00	−6.83	−4.94	−5.8
MCF/ADR	<−8.00	−7.58	−3.94	−5.5
A498	−7.52	7.51	−4.75	−6.2
Average	−7.32	7.17	−5.01	−5.9

**Table 5:** Cytotoxicity of 14 and 15 against KB cell line and resistant variants

*Compound*	*IC_50_ (nM)^a^*			
	
	*KB*	*KB-CPT*	*KB-7D*	*KB-VCR*
1	<−8.00	−8.07	−3.85	−6.5
16	<−8.00	−6.83	−4.94	−5.8
17	<−8.00	−7.58	−3.94	−5.5
14	−7.52	7.51	−4.75	−6.2
15	−7.32	7.17	−5.01	−5.9

a IC_50_ values were determined after 72 h of culturing with continuous exposure to test compounds

## Chinese Plant-derived Antineoplastic Agents and Their Analogs

Bioactivity-directed fractionation and isolation of Chinese medicinal herbs has also led to many cytotoxic lead compounds, including diterpenes (pseudolaric acids A-B,[21] kansuiphorins A-B[22]), peroxytriterpene dilactones (pseudolaride I),[23] triterpenes (polacandrin),[24] triterpene glucosides (cumingianosides A-E, cumindysosides A-B, and their modified derivatives),[25] quassinoids (bruceosides A-F),[26] sesquiterpene alkaloids (emerginatines A-B, E-F),[27,28] bisdesmosides (lobatosides B-E),[29] flavonoids (tricin and kaempferol-3-*0*-β-D-glucopyranoside)[30] and napthoquinones (psychorubin and related compounds).[31] These compounds have been reviewed previously.[1]

## Camptothecin Derivatives

The topo I inhibitor camptothecin (**16**) is a natural alkaloid isolated from the Chinese tree *Camptotheca acuminate*; it is used to treat gastric, rectal, colon, and bladder cancers.[32] Several natural and synthetic derivatives, including 9-amino (**17**)[33] and 10-hydroxy (**18**)[32] camptotecin, topotecan (**19**)[34,35] and irinotecan (**20**, CPT-11)[36,37] also are potent antitumor and DNA topo I inhibitory agents. Extensive structural modification still continues because of the limited natural availability and poor water solubility of the parent compound. To this end, we synthesized a series of water-soluble 7-(acylhydrozono)-formyl camptothecins with topo I inhibitory activity.[38] Compound **21**, containing a 7-(*L*-tyrosylhydrazono) group, was more potent than **16** in causing protein-linked DNA breaks and in inhibiting DNA topo I; however, it was less toxic in several cancer cell lines.

## Polyphenolic Compounds and Sesquiterpene Lactones

In other studies, some other classes of natural products have been found to be potent inhibitors of DNA topo II inhibitors, including polyphenolic compounds (eg, chebulinic acid, punicalagin, mallatusinic acid, acutissimin A, and sanguiin H-11),[39,40] lignans[41] and bis-(helenalinyl) (**22**) and-(isoalantodiol-B)-(**25**) glutarates.[42] The latter two compounds show >75% inhibition of DNA topo II unknotting activity at 100 µM but, unlike etoposide (**1**), do not cause DNA breakage.[42] The number of carbons in the ester linkage is important to topo II inhibition, as helenalin (**26**) itself or its malonate (**24**) or succinate (**23**) esters do not inhibit DNA topo II. However, **26** and its glutarate (**22**) ester do show similar treated/control values (162 and 195% at 8 mg/kg) in P388 leukemia-infected mice.[43]

## Antitumor Quassinoids

The bruceosides are a group of natural quassinoids isolated from *Brucea javanica*. They show selective cytotoxicity in leukemia, melanoma, and nonsmall cell lung, colon, central nervous system (CNS), and ovarian cancer cell lines.[44–46] Bruceoside C (**27**) shows excellent activity (ED_50_ < 0.1 µg/ml) in KB and RPMI-7951 cell lines. A related compound, bruceantin (**28**), has been tested in phase II clinical trials but has not progressed to drug development. Oxidation of the C-15 side chain may cause deactivation and limit the efficacy of this compound. Accordingly, we synthesized four compounds (**29** to **32**)[47] containing fluorine in the C-3 and C-15 side chains [Table 6]. The most potent compound (**29**) contained a 4,4,4-tri-fluoro-3-methyl-butanoyl ester at C-15 and was approximately as active as **28** in the eight human cancer cell lines assayed.

**Table 6:** Cytotoxicity of fluorinated quassinoids

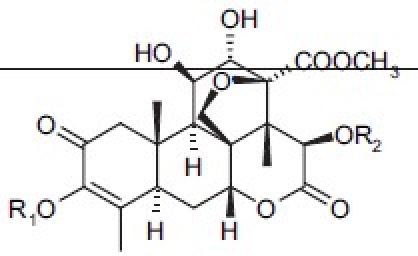			

*Compound*	*R1*	*R2*	*log GI_50_^a^*
28	H	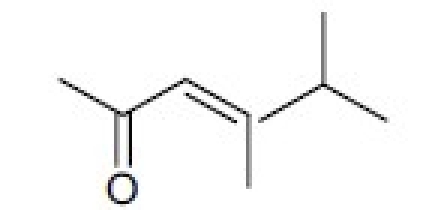	−7.7 ∼ −8.6
30	H	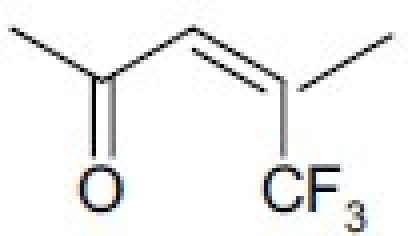	−7.0 ∼ −8.7
30	H	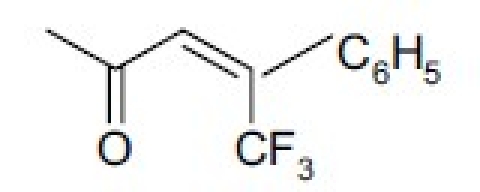	−5.0 ∼ −8.6
31	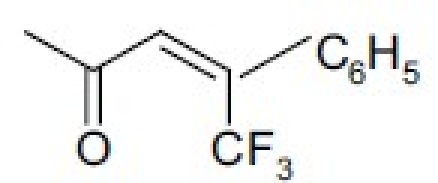	H	−4.8 ∼ −5.9
32	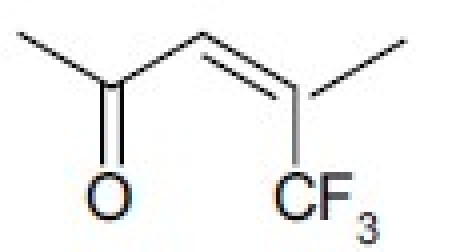	H	−4.5 ∼ −6.4

a Data from the NCI human tumor cell panel, including leukemia, nonsmall-cell lung cancer, colon cancer, CNS cancer, and others

## Flavonoid Derivatives

Other promising cytotoxic agents have been synthesized in our laboratory based on the above cytotoxic natural product models. For example, the antileukemic natural flavonoids tricin (**33**) and kaempferol-3-*0*-β-D-glucopyranoside (**34**) have percentage T/C values of 174 and 130%, respectively, at 12.5 mg/kg in P388-infected mice[30] and are structurally related to a series of synthetic cytotoxic antimitotic agents, the 2-phenyl-4-quinolones (for example, **35** and **36**). The synthetic target compounds contain a ring nitrogen instead of the oxygen found in the natural compounds. Promising activity with several of the initially synthesized 2-phenyl-4-quinolones[48] prompted the synthesis of a series of 3′,6,7-substituted compounds.[49] Several compounds showed impressive differential cytotoxicity against human tumor cell lines and were potent inhibitors of tubulin polymerization, with activity nearly comparable to that of the potent antimitotic natural products colchicine (**53**), podophyllotoxin (**3**), and combretastin A-4. The most potent compound 2-(3′methoxyphenyl)-6-pyrrolinyl-4-quinolone (**35**) had GI_50_ values in the nanomolar or subnanomolar range (average log GI_50_ = −8.73). One compound (NSC 656158) demonstrated a 130% increase in life span when tested by NCI in the xenograft ovarian OVCAR-3 model.[50]

Another structurally related series is the 2-aryl-1,8-napthyridin-4-ones (**37** and **48**, see Table 7), which contain a second nitrogen in the aromatic A ring. Compounds with *meta-*substituted phenyls (methoxy-, chloro-, or fluoro-) or á-naphthyl groups at the C-2 position showed potent cytotoxicity in the NCI 60 human tumor cell line panel with GI_50_ values in the low micromolar to nanomolar range [Table 7 and 8].[51] The tumor cell line selectivity varies with the various substituents. 2-(3′-Methoxyphenyl)napthyridinone (**37**) was significantly more cytotoxic in several cancer cell lines than the corresponding 2-(3′-methoxyphenyl)-quinolone (**36**). Both compound classes were potent inhibitors of tubulin polymerization; the 2-aryl-1,8-naphthyridin-4-ones had activity which was nearly comparable with that of the potent antimitotic natural products **53**, **3**, and combretastin A-4. Although some compounds did inhibit the binding of radiolabeled **53** to tubulin, the natural product was more potent in this assay.

**Table 7:** Antimitotic and antitumor activity of naphthyridinones 38 to 52

*Compound*	*R5*	*R6*	*R7*	*R'2*	*R'3*	*ITP^a^ IC_50_ (μM) ± SD*	*ICB^b^ % Inhibition*	*Cytotoxicity^c^ Log GI_50_*
38	CH3	H	H	H	OCH3	0.6 ± 0.1	28 ± 3	7.23
39	H	CH3	H	H	OCH3	0.80 ± 0.2	31 ± 4	7.02
40	H	H	CH3	H	OCH3	0.75 ± 0.2	29 ± 4	7.24
41	H	CH3	H	H	F	0.63 ± 02	43 ± 1	7.30
42	H	H	CH3	H	F	0.53 ± 0.8	41 ± 2	7.37
43	CH3	H	CH3	H	F	0.74 ± 0.06	29 ± 1	7.07
44	H	H	H	H	Cl	1.50 ± 0.1		6.64
45	CH3	H	H	H	Cl	1.00 ± 0.03	32 ± 1	6.80
46	H	CH3	H	H	Cl	0.72 ± 0.08	33 ± 2	6.57
47	H	H	CH3	H	C1	0.89 ± 0.09	38 ± 1	6.77
48	CH3	H	CH3	H	C1	0.77 ± 0.2	22 ± 2	6.46
49	H	H	H	CH = CH-H = H		1.10 ± 0.3		7.45
50	CH3	H	H	CH = CH-H = H		0.93 ± 0.2	37 ± 4	7.45
51	H	CH3	H	CH = CH-H = H		0.55 ± 0.05	46 ± 3	7.72
52	H	H	CH3	CH = CH-H = H		0.66 ± 0.1	40 ± 4	7.18
Colchicine (53)						0.80 ± 0.07		7.24
Podophyllotoxin (3)						0.46 ± 0.02		7.24

a ITP = Inhibition of polymerization;

b ICB = inhibition of colchicine binding;

c data are average values from over 60 human tumor cell lines, including leukemia, nonsmall cell and small cell lung cancer, colon cancer, CNS cancer, ovarian cancer, and renal cancer

**Table 8:** Total inhibition of in vivo tumor cell growth by 2-(3′-Halophenyl)-1,8-Naphthyridine-4-ones 41 to 48^a^

*Cell type*	*Cytotoxicity [log TGI (M)]^b^*							
	
	*41*	*42*	*43*	*44*	*45*	*46*	*47*	*48*
Leukemia	−5.57	−5.56	−5.61	−4.41	<−4.00	−4.14	<−4.00	−4.09
Non small cell lung cancer	−4.79	−5.24	−5.60	−4.07	<−4.00	−4.35	−4.61	<−4.00
Colon cancer	−6.49	−6.26	−5.93	−4.79	−4.92	−5.02	−5.51	−4.54
CNS cancer	−5.51	−5.65	−5.01	−4.78	−4.74	−5.72	−5.71	−5.30
Melanoma	−4.49	−4.62	−4.86	−4.01	−4.15	4.32	−4.16	−4.14
Ovarian cancer	−4.57	−4.99	−5.26	−4.50	−4.56	4.80	−4.89	−4.52
Renal cancer	−4.26	−4.19	−4.31	−4.31	−4.16	4.06	<−4.00	−4.23
Prostate cancer	−6.16	−5.80	−4.31	−5.58	−5.63	<−4.00	<−4.00	−5.51
Breast cancer	−6.27	−6.24	−6.00	−5.93	−6.09	−4.89	−5.42	−5.91

a Data obtained from the in vitro disease human tumor calls screen;

b Log molar concentrations required to cause total growth inhibition

## Colchicine Derivatives

Colchicine (**53**), an alkaloid isolated from *Colchicum autumnale,* is one of the oldest drugs still in use. It is used to treat gout and familial Mediterranean fever. It has potent antitumor activity against P388 and L1210 mouse leukemia, which is related to its powerful antimitotic effects. Colchicine binds to and inhibits the polymerization of tubulin, which plays an essential role in cellular division. The synthetic analog thiocolchicine (**54**) is more potent and more toxic than **53**; the corresponding IC_50_ values for inhibition of tubulin polymerization (ITP) are 0.65 and 1.5 µM, respectively.[52]

Because the toxicity of **53** and **54** limits their medicinal value, structural modification is directed toward creating less toxic and more selective antimitotic analogs. Through the synthetic routes shown in Scheme 1.1, analogs of **54** were prepared with ketone (**55**, thiocolchicone), hydroxyl (**56**), and ester (**57**, **58**) groups replacing the C-7 acetamido group.[53] Chromatographic separation followed by hydrolysis of diastereoisomeric camphanate esters allowed preparation of both enantiomeric alcohols and esters. Only the (-)-aS,7S optically pure enantiomers [the C-7 alcohol, (-)-**56**, and its acetate, (-)-**57**, and isonicotinoate, (-)-**58**, esters] showed activity (ITP IC_50_ values ranging from 0.56 to 0.75 µM) equivalent to or greater than that of (-)-**54**. Reacting thiocolchicone (**55**) with aniline caused contraction of the seven-membered C-ring, producing the alloketone (**59**) deaminodeoxy-colchinol-7-one thiomethyl ether.[54] This compound also showed antimitotic activity comparable with that of **55**.

## Quinone Derivatives

Many naturally occurring substituted anthraquinones [including morindaparvin-A (**60**) and morindaparvin-B (**61**)] and napthaquinones (including psychorubin and related compounds) possess cytotoxic antileukemic activities.[55–57] In the former compounds, removing the hydroxyl substituents retained or increased cytotoxicity; for example, **62** lacks one hydroxyl (R4 = H) found in **61** (R4 = OH) and is more active in the KB cell line (ID_50_: **61**, 4.0 mg/kg; **62**, 0.09 mg/kg).

The anthraquinone mitoxanthrone (**63**) is a clinically useful antineoplastic agent. This compound contains a planar chromophore that could potentially insert or intercalate between DNA base pairs, a feature frequently found in antineoplastic agents. Alkylating agents (eg, cyclophosphamide and busulfan) are another class of antineoplastic drugs. This large, diverse group of compounds contains reactive groups that are capable of covalently modifying a variety of biological molecules. For example, teroxirone (**64**) is a 1,3,5-triazine with alkylating epoxide moieties in its amino side chains, and has been reported to exhibit antineoplastic activity.[58] The known anthraquinone 1,4-bis-(2,3epoxypropylamino)-9,10-anathracenedione (**65**) contains both the planar skeleton and diamino side chain substitution pattern of **63** and the alkylating epoxide moiety of **64**. In preliminary *in vitro* studies, **65** showed significant and selective activity with an ED_50_ of less then 40 ng/ml against human epidermoid carcinoma (KB cells). Based on this promising result, a SAR study was implemented.[59] Derivatives of **65** containing alkene, epoxide, halohydrin, diol, and secondary amine functional groups in the alkylamino side chains and with quinine or napthoquinone skeletons were prepared and tested for *in vitro* antineoplastic activity. The results showed that, in general, analogs with no alkyl side chains, alkene, secondary amines, diols, or side chains containing four instead of three carbons were less cytotoxic, while compounds containing alkylating epoxide or halohydrin moieties exhibited greater activity. Hydroxy substitution on the planar skeleton in conjunction with alkylating side chains produced compounds with the most potent cytotoxic activity. Activity was retained when the amine linkage in the parent compound was replaced by an ether linkage, giving 1,4-bis-(2,3-epoxypropoxy)-9,10-anthracenedione (**66**). Both of these compounds showed excellent activity in leukemia and melanoma cell lines [Table 9].

**Table 9:** Cytotoxicity of quinines 65 and 66 in selected cell lines

*Cell line*	*Log GI_50_ (M)*	
	
	*65*	*66*
Leukemia		
CCRF-CEM	−8.00	−8.00
HL-60 (TB)	−8.00	−7.34
MOLT-4	−8.00	−7.81
Nonsmall cell-lung cancer		
NCI-H23	−6.10	−6.10
NCT-116	−6.77	−6.29
CNS cancer-SF-268	−7.06	−6.52
Melanoma-LOXIMVI	−8.00	−7.56
ovarian cancer-OVCAR-8	−6.16	−5.40
Renal cancer-ACHN	−6.66	−6.46
Prostate cancer-DU-145	−5.63	−6.34
Breast cancer-MCF-7	−7.30	−6.88

**Scheme 1:**
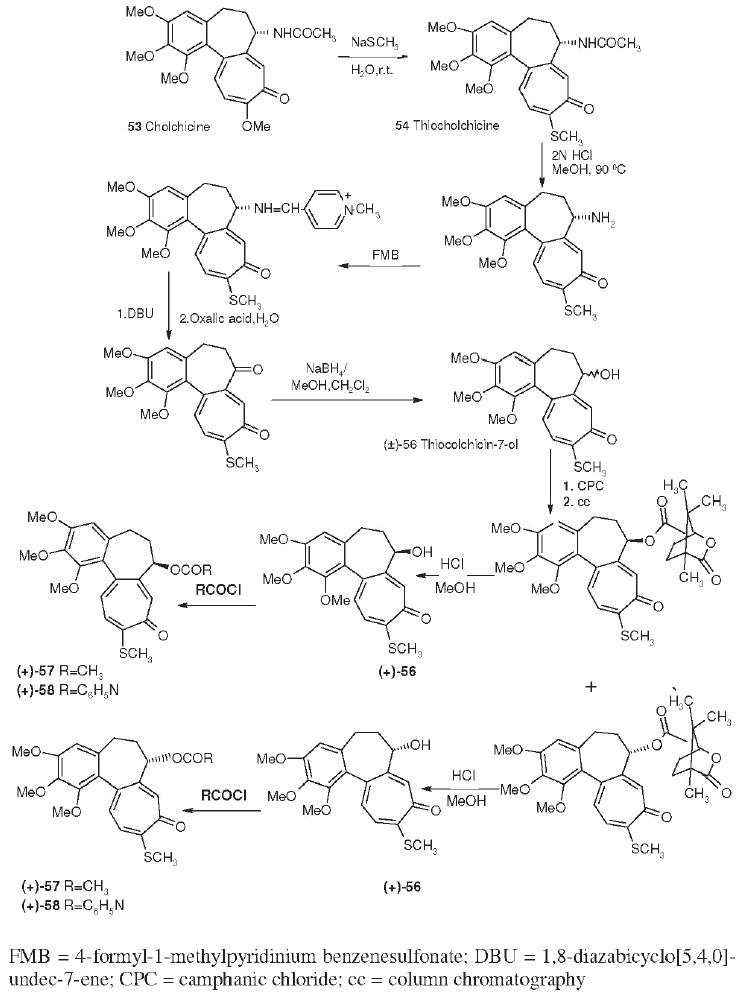
Synthesis of thiocolchicine, (+) and (−) deacetamidothiocolchicine-7-oland esters

**Scheme 2:**
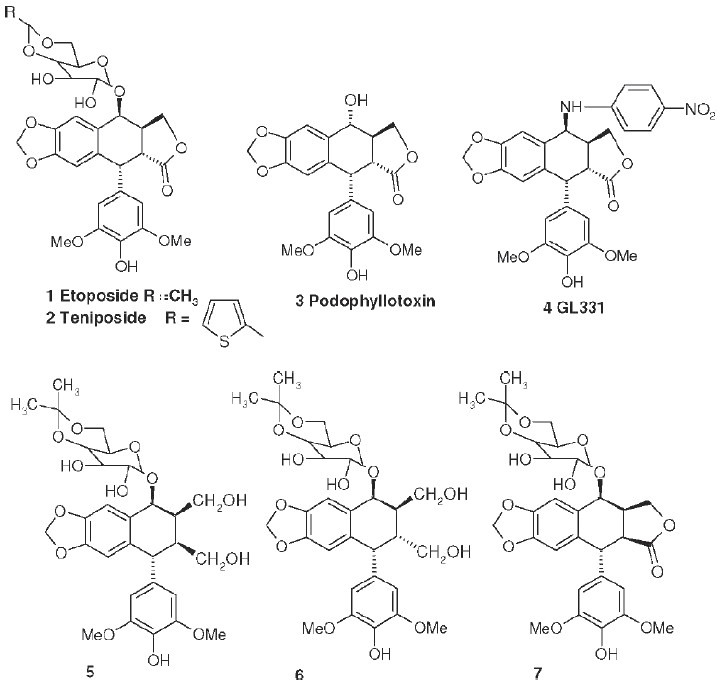


**Scheme 3:**
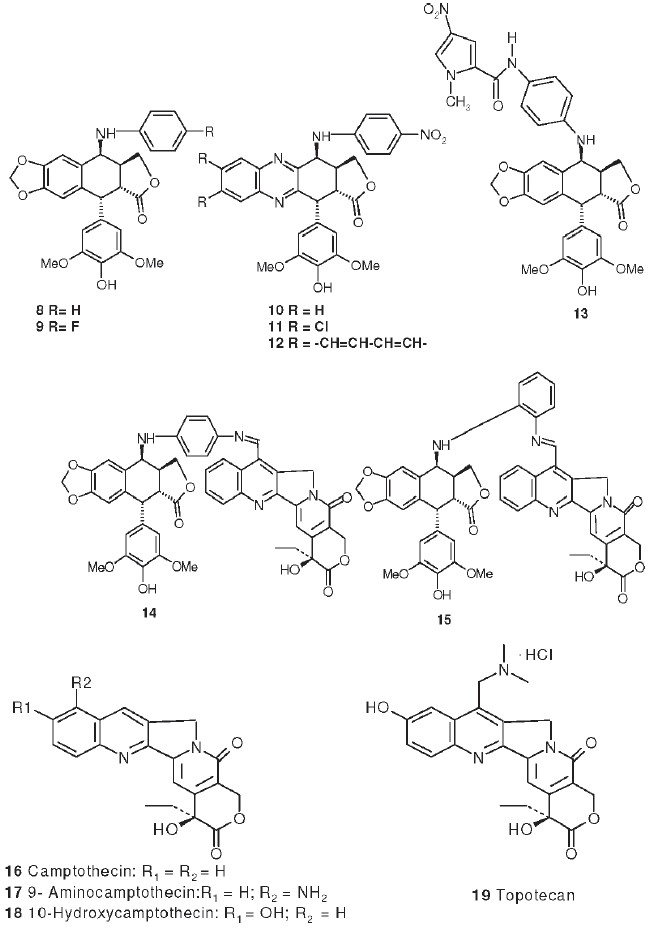


**Scheme 4:**
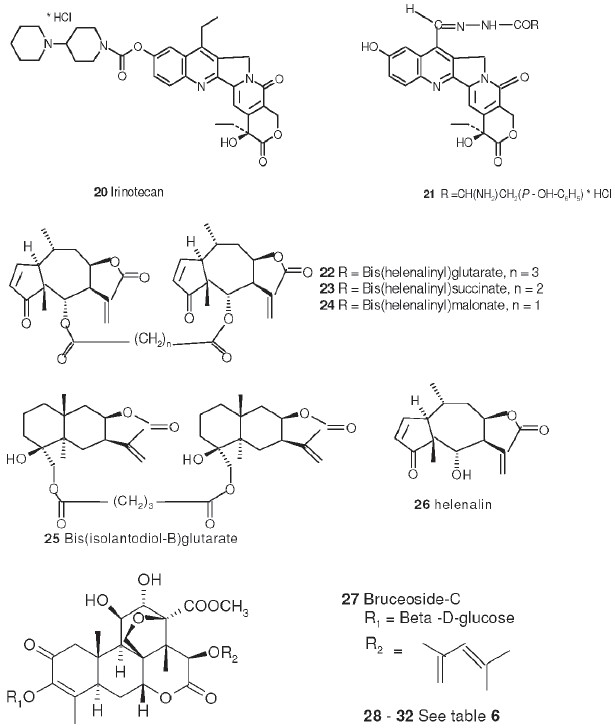


**Scheme 5:**
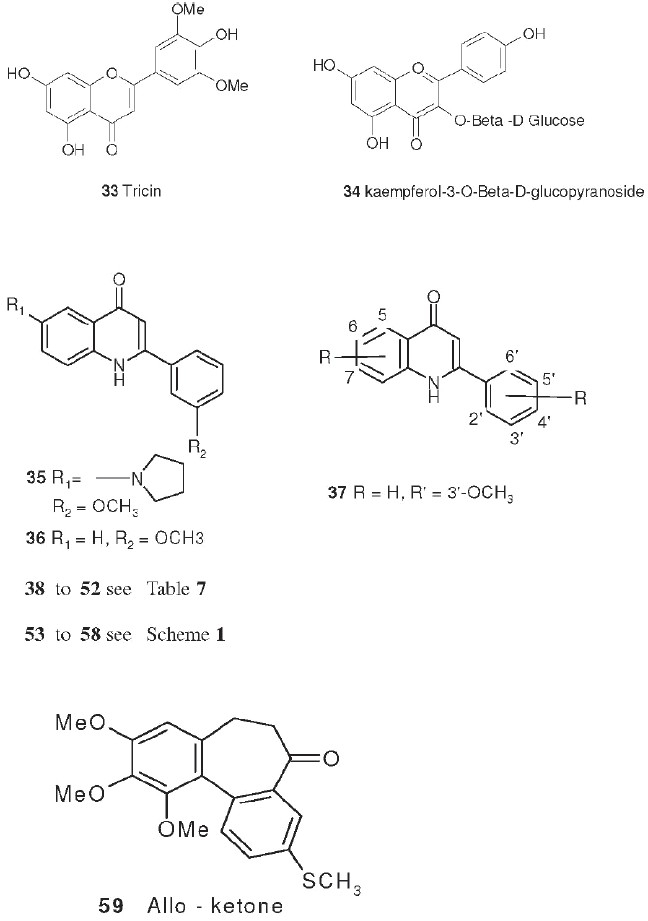


**Scheme 6:**
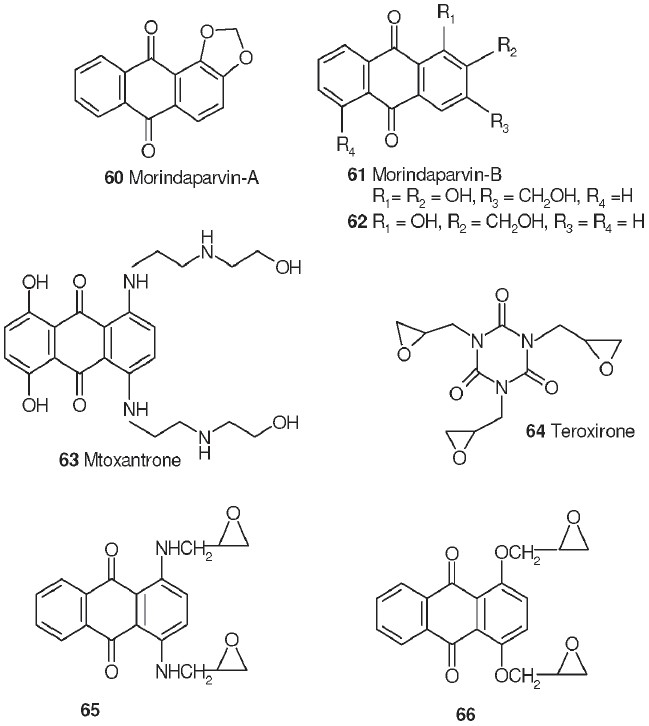


## Conclusion

In the continuing search for potentical anticancer agents, GL331 (**4**), which is currently in phase IIa clinical trials, highlights the current study. However, over the last several years, more than 100 new cytotoxic antitumor compounds and their analogs have been found with confirmed activity in the NCI *in vitro* human tumor cell lines bioassay. These compounds are of current interest of NCI for further *in vivo* evaluation and to us for further lead improvement and drug development. Based on this successful identification of plant-derived antitumor drug candidates, we can look forward to further successes in this research area in the future.
